# The complete chloroplast genome sequence of *Picrasma quassioides* (D. Don) Benn. 1844 (Simaroubaceae)

**DOI:** 10.1080/23802359.2022.2087545

**Published:** 2022-06-23

**Authors:** Liu Qin, Geng Xiaoshan, Yipeng Guo, Huang Huaxi, Zhang Huiye, Yulin Zhu, Rong Chen

**Affiliations:** a Guangxi Key Laboratory of Agricultural Resources Chemistry and Biotechnology, Yulin Normal University, Yulin, PR China; b Hutchison Whampoa Guangzhou Baiyunshan Chinese Medicine Co., Ltd, Guangzhou, PR China

**Keywords:** *Picrasma quassioides*, chloroplast genome, Simaroubaceae, phylogeny

## Abstract

*Picrasma quassioides* is a member of the Simaroubaceae family and is widely used as a medicinal plant. In this study, we sequenced and assembled the complete chloroplast genome of *P. quassioides*. The chloroplast genome is 160,015 bp in length, with a large single-copy region of 87,136 bp, a small single-copy region of 18,069 bp, and a pair of inverted repeat regions of 27,405 bp. It contains a total of 110 unique genes, including 77 protein-coding genes, 29 tRNA genes, and 4 rRNA genes. Phylogenetic analysis showed that *P. quassioides* clustered well with Simaroubaceae plants, *Eurycoma longifolia*, *Leitneria floridana*, and *Ailanthus latissimus*.

*Picrasma quassioides* (D. Don) Benn. 1844, also known as ‘nigaki’ bitterwood, is a kind of small tree or shrub that belongs to the Simaroubaceae family, which is mainly distributed in tropical and subtropical Asia (Alves et al. 2014). As a traditional Chinese medicinal plant, *P. quassioides* has anti-inflammatory, anti-malarial, anti-cancer, and antihypertensive properties (Bai et al. 2020). The crude extract of *P. quassioides* contains a variety of beneficial phytochemicals, such as alkaloids and polyphenols, which have a wide range of pharmacological activities, from anti-inflammatory to anti-parasitic. So far, most studies on *P. quassioides* have focused on its chemical composition and pathology, with little attention paid to its molecular biology and evolution. Therefore, in this study, we sequenced, assembled, annotated, and analyzed the phylogenetic tree of the complete chloroplast genome of *P. quassioides* to increase our understanding of the phylogenetic and evolutionary history of the species.

The green leaves of *P. quassioides* were collected from Yulin Normal University (110.183°E, 22.660°N) in Yulin, Guangxi, China. The voucher specimen (KM-YNU-001) was deposited in the herbarium of Yulin Normal University (https://syy.ylu.cn/index.html, Yulin Zhu, gxzyl@163.com). All the research met ethical guidelines and adhered to the legal requirements of the study country. An enhanced CTAB technique was applied to extract total genomic DNA. The Illumina Hiseq 4000 platform (150 bp * 2) (Biozeron, Shanghai, China) was utilized for the sequencing. Trimmomatic (Bolger et al. 2014) was used to remove adapters and low-quality reads from the raw data using the following settings: ILLUMINACLIP 2:30:10, LEADING:3, TRAILING:3, SLIDINGWINDOW:4:25, and MINLEN:85. The GetOrganelle toolkit (Jin et al. 2020) and CPGAVAS2 (Shi et al. 2019) were used to accomplish the *de novo* assembly and chloroplast genome annotation using the default parameters. The cp sequence of *P. quassioides* was submitted to NCBI, and the accession number was MZ902043.

The chloroplast genome of *P. quassioides* is 160,015 bp in length, with a large single-copy region (LSC) of 87,136 bp, a small single-copy region (SSC) of 18,069 bp, and two inverted repeat regions (IRs) of 27,405 bp. The overall GC content of the *P. quassioides* cp genome was 37.96%, with the LSC, SSC, and IR regions accounting for 36.16%, 32.39%, and 42.67%, respectively. The cp genome of *P. quassioides* had 110 distinct genes, including 77 protein-coding genes, 29 tRNA genes, and 4 rRNA genes. Eighteen of these 110 genes had introns, including eight tRNA genes and ten protein-coding genes. The genes *ycf3* and *clpp* had two introns, while the remaining 16 genes only had one intron.

To confirm the phylogenetic position of *P. quassioides* and further clarify the evolutionary relationships within the family Simaroubaceae, phylogenetic analyses based on a total of 23 complete cp genomes that belong to the order Sapindales were performed. *Manihot esculenta*, which belongs to the family Euphorbiaceae, was selected as the outgroup. After aligning the cp genomes with MAFFT (Rozewicki et al. 2019) and removing gaps with Gblocks (Castresana 2000), a Maximum Likelihood (ML) phylogenetic tree was built with IQ-TREE (Nguyen et al. 2015) with bootstrap 1000 repetitions and the TVM + F + I + G4 model was chosen (Figure 1). In the tree, *P. quassioides* was the sister group of *Eurycoma longifolia*, *Leitneria floridana*, and *Ailanthus latissimus* with high bootstrap support (100%). This result supports the fact that all those four species belong to the Simaroubaceae family.

**Figure 1.**
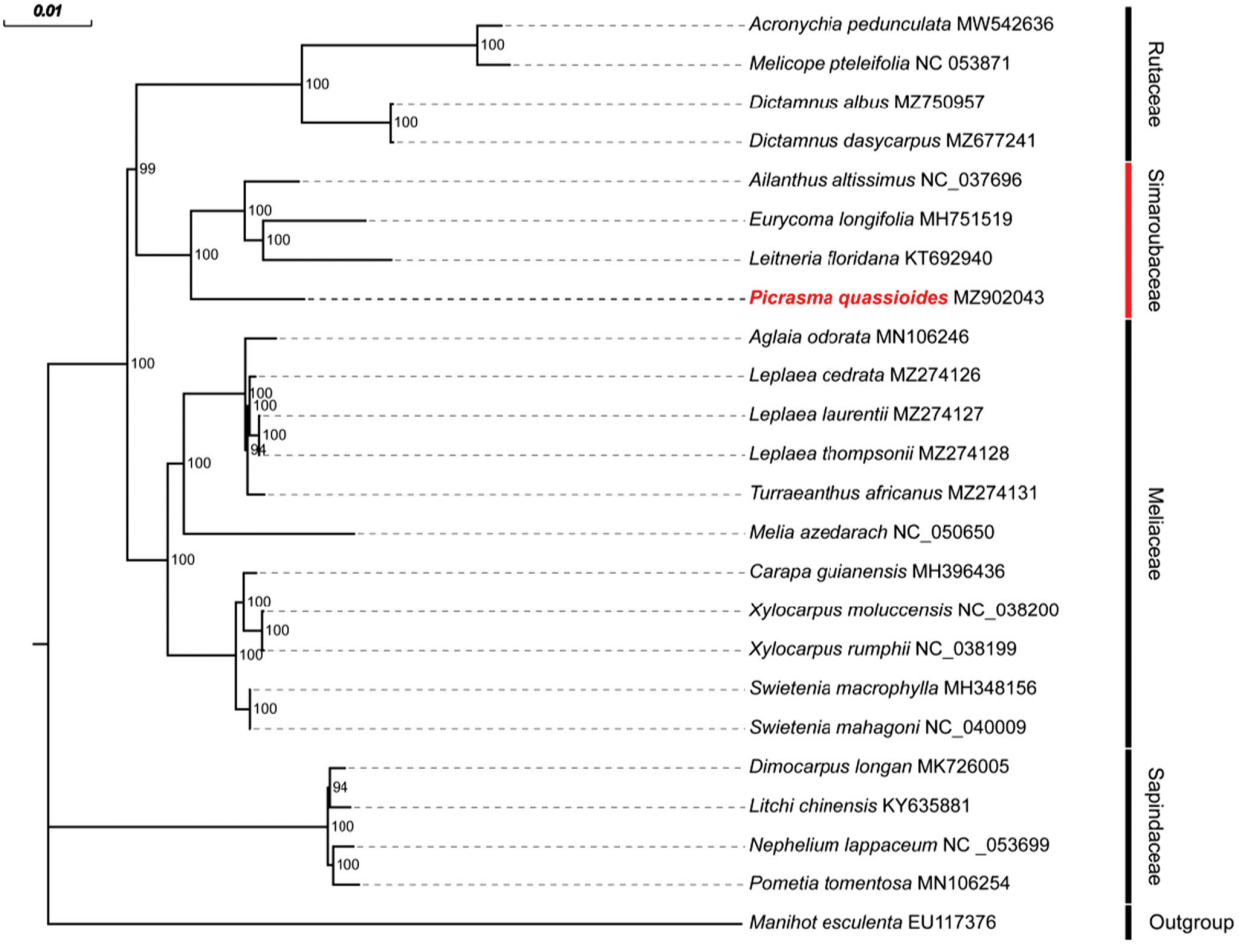
Maximum-likelihood phylogenetic tree based on all protein-coding genes in the cp genomes of 23 Sapindales species. Bootstrap values (1000 replicates) are shown at the nodes.

## Data Availability

The data that support the findings of this study are openly available in GenBank of NCBI at https://www.ncbi.nlm.nih.gov, under the accession number MZ902043. The associated BioProject, SRA, and BioSample numbers are PRJNA762200, SRR15839410, and SAMN21380154, respectively.

## References

[CIT0001] Alves IABS, Miranda HM, Soares LAL, Randau KP. 2014. Simaroubaceae family: botany, chemical composition and biological activities. Rev Bras Farmacogn-Braz J Pharmacogn. 24(4):481–501.

[CIT0002] Bai M, Zhao WY, Xu W, Zhang YY, Huang XX, Song SJ. 2020. Triterpenoids from Picrasma quassioides with their cytotoxic activities. Phytochem Lett. 39:128–131.

[CIT0003] Bolger AM, Lohse M, Usadel B. 2014. Trimmomatic: a flexible trimmer for Illumina sequence data. Bioinformatics. 30(15):2114–2120. 24695404 10.1093/bioinformatics/btu170 PMC4103590

[CIT0004] Castresana J. 2000. Selection of conserved blocks from multiple alignments for their use in phylogenetic analysis. Mol Biol Evol. 17(4):540–552. 10742046 10.1093/oxfordjournals.molbev.a026334

[CIT0005] Jin JJ, Yu WB, Yang JB, Song Y, dePamphilis CW, Yi TS, Li DZ. 2020. GetOrganelle: a fast and versatile toolkit for accurate de novo assembly of organelle genomes. Genome Biol. 21(1):241. 32912315 10.1186/s13059-020-02154-5 PMC7488116

[CIT0006] Nguyen LT, Schmidt HA, Von Haeseler A, Minh BQ. 2015. IQ-TREE: a fast and effective stochastic algorithm for estimating maximum-likelihood phylogenies. Mol Biol Evol. 32(1):268–274. 25371430 10.1093/molbev/msu300 PMC4271533

[CIT0007] Rozewicki J, Li S, Amada KM, Standley DM, Katoh K. 2019. MAFFT-DASH: integrated protein sequence and structural alignment. Nucleic Acids Res. 47(W1):W5–W10. 31062021 10.1093/nar/gkz342 PMC6602451

[CIT0008] Shi L, Chen H, Jiang M, Wang L, Wu X, Huang L, Liu C. 2019. CPGAVAS2, an integrated plastome sequence annotator and analyzer. Nucleic Acids Res. 47(W1):W65–W73. 31066451 10.1093/nar/gkz345 PMC6602467

